# The effect of lipid accumulation product and its interaction with other factors on hypertension risk in Chinese Han population: A cross-sectional study

**DOI:** 10.1371/journal.pone.0198105

**Published:** 2018-06-06

**Authors:** Jian Song, Yingying Zhao, Sumei Nie, Xue Chen, Xuesen Wu, Jing Mi

**Affiliations:** 1 School of public health, Bengbu medical college, Bengbu, Anhui Province, China; 2 Bengbu health board, Bengbu, Anhui Province, China; Beijing Key Laboratory of Diabetes Prevention and Research, CHINA

## Abstract

**Objectives:**

Lipid accumulation product (LAP) is a simple and effective indicator that reflects visceral obesity. This study aimed to compare the significance of LAP in predicting hypertension risk with other obesity indices, and to evaluate the interactive effects of LAP and smoking, family history of hypertension on hypertension risk in Chinese Han adults.

**Methods:**

A community based cross-sectional study was performed in Bengbu, China. Participants received face-to-face questionnaire survey, anthropometric tests and laboratory examinations. Relevant indicators that reflect obesity including BMI (body mass index), waist-to-height ratio (WHtR) and LAP were calculated. Multivariate logistic regression analysis was applied to explore the association between LAP and hypertension risk. The area under the receiver-operating characteristics curves (AUC) of LAP, BMI, and WHtR were calculated and then compared. Interactive effect was evaluated by relative excess risk due to interaction (RERI), attributable proportion due to interaction (AP) and synergy index (SI).

**Results:**

A total of 1777 participants were enrolled, and the prevalence of hypertension was 24.4% (n = 433). There was a significant increase in hypertension risk with LAP levels in the fourth quartile as compared with the bottom quartile (OR: 3.31, 95%CI: 1.76–6.25). The AUC of LAP was significantly different than that of BMI in males (*Z* = 2.158, *p* = 0.0309) and females (*Z* = 3.570, *p* = 0.0004), while only performed better in females as compared with that of WHtR (*Z* = 2.166, *p* = 0.0303). LAP was significantly interacted with family history of hypertension on hypertension risk both in males (RERI: 1.07, 95%CI: 0.09–2.05; AP: 0.33, 95%CI: 0.23–0.44; SI: 1.92, 95%CI: 1.53–2.41) and females (RERI: 0.80, 95%CI: 0.07–1.53; AP: 0.25, 95%CI: 0.11–0.39; SI: 1.59, 95%CI: 1.16–2.19). However, a significant interaction between LAP and smoking was only observed in males (RERI: 1.32, 95%CI: 0.15–2.75; AP: 0.40, 95% CI: 0.14–0.73).

**Conclusion:**

Increased LAP was significantly associated with a higher risk of hypertension in Chinese Han adults. Moreover, the effect of LAP on predicting hypertension risk was better than that of other obesity indices. Our results also demonstrated interactive effects of LAP with smoking, family history of hypertension on hypertension risk.

## Introduction

Hypertension is one of the most serious public health issues worldwide with an increased prevalence in recent years [1]. It was reported that 27.8% of Chinese adults were hypertensive [2]. Moreover, hypertension is also a predominant risk factor for cardiovascular diseases [3]. A prospective cohort study with 500223 adults in China indicated that hypertension accounted for about one-third of deaths due to cardiovascular diseases [4].

The prevalences of obesity and obesity-related diseases have dramatically increased both in developing and developed countries [5, 6]. From 2007 to 2013, the age-standardized prevalence of obesity in Northeastern China increased from 15.82% to 19.41% in males and 13.18% to 18.77% in females, respectively [7]. Traditionally, body mass index (BMI) and waist-to-height ratio (WHtR) are most frequently used indices to evaluate general obesity and abdominal obesity [8]. BMI can reflect the degree of overweight, but cannot reflect the individual fat distribution. Relevant studies have suggested that abdominal fat distribution may be more closely related to adverse outcomes than those of BMI, such as cardiovascular diseases [9, 10]. However, WHtR only reflects abdominal obesity accurately, but cannot distinguish between subcutaneous fat and visceral fat.

Recently, visceral obesity has attained increasing attention because of its higher value in predicting diseases risks [11, 12]. Also, emerging evidences have suggested that visceral fat may be more closely associated with hypertension risk. Compared with subcutaneous fat, visceral fat is the predominant cause of insulin resistance, dyslipidemia and cardiovascular diseases [13, 14]. Visceral adipose tissue can activate the renin-angiotensin system by releasing angiotensinogen, angiotensin converting enzyme and cathepsin [15]. Moreover, visceral fat expressed more angiotensinogen and more proinflammatory cytokines than that of subcutaneous adipose tissue [16]. Meanwhile, visceral fat was reported to be related with increased activity of sympathetic nervous system, which was also associated with hypertension [17]. Computer tomography(CT) and magnetic resonance imaging (MRI) are the gold standards to evaluate visceral fat in clinical application, their high costs and radiation exposure, however, significantly limit their widely use in practice. Therefore, searching for a simple and effective indicator reflecting visceral obesity is urgent.

LAP, as the product of waist circumference (WC) and triglycerides (TG), was proposed as a simple and effective index for lipid over accumulation among adults by Kahn et al [18]. LAP can better reflect the total fat body accumulation and visceral fat function, rather than simple high body weight. The third National Health and Nutrition Examination Survey III showed that LAP performed better than that of BMI for identifying higher total cholesterol, low-LDL-C, uric acid levels, higher total cholesterol/HDL-C and lower HDL-C levels among US adults [18]. Several studies have suggested that LAP can better predict metabolic syndrome, insulin resistance and diabetes risks [19–21]. In a cross-sectional study in Japan, LAP was suggested to be better for discriminating the risk of hypertension [22]. Gao et al [23] compared the ability of BMI and LAP in predicting hypertension risk among Mongolians, and the results showed that the performance of LAP performed was superior to that of BMI. As the ethnicity differences of body composition, the value of LAP in Han Chinese adults remains unclear. Chinese individuals have a greater amount of visceral adipose tissue than Europeans at a given BMI or WC [24]. Therefore, the relationship between LAP and hypertension risk in the Han of China needs to be further confirmed. Additionally, hypertension is regarded as a multifactorial disease that is associated with genetic and environmental factors. Besides, the interactions of gene-environment and environment- environment may aggravate the risk of hypertension. Previous studies have indicated that smoking and family history of hypertension were related with hypertension risk [25, 26]. To the best of our knowledge, there was no article exploring the interactive effects of LAP and smoking, family history of hypertension on hypertension risk.

In the present study, we first evaluated the association between LAP and hypertension risk in Chinese Han adults. Secondly, the abilities of BMI, WHtR and LAP in predicting hypertension risk were compared. Finally, we assessed the interactive effects between LAP and smoking, family history of hypertension on hypertension risk.

## Materials and methods

### Study participants

A community based cross-sectional survey was conducted in Longzihu, Bengbu, China. Firstly, seven communities were selected by a stratified sampling. Then, simple random sampling was used to identify participants. Inclusion criteria: (1) live in the selected communities for more than 6 months in the past year; (2) middle-aged and elderly adults; (3) willingness to participate in this project. Exclusion criteria: (1) have no abilities to normally communicate with investigators due to psychological or mental barriers; (2) cannot finish the overall survey independently because of inconvenience or serious illness. Written informed consent was obtained from each participant. The overall survey had three parts: face-to-face questionnaire, anthropometric tests and laboratory examinations. This study was approved by the Ethics Committee of Bengbu medical college.

### Questionnaire survey

We used a self-designed questionnaire to investigate relevant information for each participant by sophisticated members through face-to-face interviews. Among them, smoking was defined as the status of pre-smoking or current-smoking. Educational level was classified as “elementary school or lower”, “middle school graduate” and “high school graduate or higher”. Marital status was categorized as “currently not married” and “currently married”. Family income was grouped as “0–2000”, “2000–4000” and “4000-”. Participants were required to answer the question “do you have a family history of hypertension (yes or no)”. Positive family history of hypertension was defined as at least one parent or sibling with hypertension.

### Anthropometric tests and laboratory examinations

Height and weight were measured with the participants in lightweight clothing and without shoes. Waist circumference (WC) was measured at the level midway between the lower rib margin and the iliac crest. Blood pressure was detected using mercury sphygmomanometer by trained members according to standardized methods [27]. All subjects were required to have a rest for about 10 mins before measuring blood pressure in a quiet environment. Blood samples were collected from the antecubital vein after an overnight fast. Routine biochemical data including fasting plasma glucose (FPG), triglycerides (TG) and HbA1c were examined. FPG≥7.0 mmol/L, TG≥1.70mmol/l and HbA1c≥6.5% were defined as hyperglycemia, hypertriglyceridaemia and hyper-HbA1c, respectively [28, 29].

### Definitions

1. Hypertension was defined as systolic blood pressure (SBP)≥140 mmHg, or diastolic blood pressure (DBP)≥90 mmHg, or the subject reported with a medical history of anti-hypertensive medication [30].
2. BMI was referred to weight (kg)/height (m)^2^. According to the Working Group on Obesity in China [31], BMI≥ 28 was defined as general obesity.
3. WHtR was calculated by dividing WC by height, and ≥0.5 was defined as abdominal obesity [32].
4. LAP was calculated as [WC (cm)-65]by height Obesity ales, and [WC (cm)-58]×[TG(mmol/L)] in females [18].

### Statistical analysis

All data were entered into Epidata 3.1 software firstly by using double entry approach. Quantitative data were presented as meansentede data were into Epidata 3.1 software firstly by using double entry approach. bioparticipants were compared by *t*-test for normally distributed data or Wilcoxon rank sum test for non-normally distributed data. LAP was divided into four groups (Q1, Q2, Q3, and Q4) by quartiles. The differences of quantitative data across the LAP groups were compared by analysis of variance if the data were normally distributed and homogeneity of variance. Otherwise, Kruskal-Wallis H test was used. Categorical variables were expressed as percentages, and compared by Chi-squared test. Multivariate logistic regression model was performed when analyzing the relationship between LAP and hypertension risk. Optimal cut-off values of BMI, WHtR and LAP in predicting hypertension were identified according to best Youden index (YI, sensitivity+specificity−1). The area under the receiver-operating characteristics (ROC) curves (AUC) of LAP, BMI, and WHtR were calculated, and then compared by non-parametric significance test (statistic of *Z*). Finally, the interactive effects between LAP and family history of hypertension, smoking on risk of hypertension were examined by relevant indicators including the relative excess risk due to interaction (RERI = RR_11_-RR_10_-RR_01_+1), the attributable proportion due to interaction (AP = RERI/RR_11_), and the synergy index (SI = (RR_11_-1)/(RR_01_-1)+(RR_10_-1)). All of these indicators were calculated using the Excel table designed by Andersson et al [33, 34]. The interactive effect was considered as statistically significant if the corresponding 95% CI for RERI, AP, and SI did not overlap 0, 0 and 1, respectively. All *p* values were two-sided and *p*<0.05 was considered as statistically significant. Statistical calculations were performed using SPSS19.0 and Medcalc software.

## Results

### 1. Basic characteristics

There were 1777 participants (748 men and 1029 women) with the average age of 60.82 years enrolled in this study. The overall prevalence of hypertension was 24.4% (n = 443). Male participants had a higher prevalence of hypertension than that of female (*p*<0.001). For the anthropometric measurements, there were statistically significant differences for BMI, WHtR and LAP between hypertension and non-hypertension members (*p*<0.001). TG (*p*<0.001), FPG (*p*<0.001) and HbA1c (*p* = 0.002) were significantly higher in hypertension participants. Significant differences in educational level (*p* = 0.017), family history of hypertension (*p* = 0.003) and smoking status (*p* = 0.012) between hypertension and non-hypertension members were also observed. However, the differences in marital status (*p* = 0.428) and family income (*p* = 0.673) were not statistically significant. The basic characteristics of participants were shown in Table 1.

**Table 1:** Basic characteristic of the study participants.

Variables	Non-hypertension(N = 1344)	Hypertension (N = 433)	t/χ^2^/Z	*P*
Gender (male %)	39.80	49.19	11.834	0.001
Age (years)	60.33±11.38	62.31±10.64	-3.222	0.001
Educational level			8.197	0.017
Elementary level or lower (%)	31.40	38.80		
Middle school graduate (%)	36.98	33.72		
High school graduate or higher (%)	31.62	27.48		
Marital status (currently married %)	83.90	85.45	0.629	0.428
Family income (yuan)			0.791	0.673
0–2000 (%)	54.61	53.12		
2000–4000 (%)	40.33	42.73		
>4000 (%)	5.06	4.16		
BMI(kg/m^2^)	24.49±4.08	25.85±3.70	7.981	<0.001
WHtR	0.52±0.06	0.56±0.06	10.518	<0.001
Hyperglycemia (%)	9.60	16.40	15.157	<0.001
Hypertriglyceridaemia (%)	32.36	48.27	35.839	<0.001
LAP	41.95±29.24	63.82±41.02	11.437	<0.001
Hyper-HAb1c (%)	15.48	21.94	9.674	0.002
Smoking (%)	28.05	34.41	6.358	0.012
Family history of hypertension (%)	16.90	23.33	8.971	0.003

### 2. LAP and hypertension risk

LAP was grouped by quartiles in Table 2. Male had a relatively higher LAP than that of female (*p*<0.001). Participants with higher LAP quartiles had significantly higher BMI (*p*<0.001) and WHtR (*p*<0.001). The prevalence of hypertension (*p*<0.001), hyperglycemia (*p*<0.001), hyper-HbA1c (*p*<0.001), smoking (*p* = 0.043) progressively increased across LAP quartiles. However, family history of hypertension (*p* = 0.761), age (*p* = 0.347), marital status (*p* = 0.105), educational level (*p* = 0.157) and family income (*p* = 0.112) had no significant differences across LAP quartiles.

**Table 2:** The comparisons of cardiovascular risk factors according to the quartiles of LAP.

Variables	LAP				F/H/χ^2^	*P*
	Q1(<23.6) Q2(23.6–38.1) Q3(38.1–61.7) Q4(>61.7)					
Number of participants	444	446	443	444	-	-
Age (years)	60.43±11.90	60.99±11.08	61.53±11.29	60.32±10.66	3.305	0.347
Gender (male %)	32.21	42.15	43.57	50.45	30.920	<0.001
BMI	22.18±3.18	24.48±3.60	25.56±3.24	27.08±4.35	473.571	<0.001
WHtR	0.48±0.04	0.52±0.05	0.55±0.05	0.58±0.06	623.872	<0.001
Hyperglycemia (%)	4.73	8.30	11.29	20.72	62.667	<0.001
Hyper-HbA1c (%)	9.68	12.11	19.19	27.25	58.838	<0.001
Hypertension (%)	9.46	20.63	26.86	40.54	121.441	<0.001
Family history of hypertension (%)	18.02	20.18	17.83	17.79	1.165	0.761
Smoking (%)	25.90	29.37	28.67	34.46	8.143	0.043
Marital status (currently married %)	84.68	83.41	81.49	87.39	6.136	0.105
Educational level (high school graduate or higher %)	31.76	33.41	30.70	26.58	9.300	0.157
Family income (>4000%)	2.93	4.28	6.12	4.97	10.310	0.112

We then analyzed the relationship between LAP and hypertension risk by logistic regression model. The crude OR was 6.35 (95%CI: 4.39–9.12) of LAP levels in the fourth quartile as compared with the first quartile. A significant increase in hypertension risk with LAP levels in the fourth quartile as compared with the first quartile was also observed by multivariate analysis (adjust OR:3.31, 95% CI: 1.76–6.25). The results were presented in Table 3.

**Table 3:** OR (95%CI) of LAP on risk of hypertension by logistic regression mode.

Quartiles	Number of hypertension cases	OR^1^(95%CI)	OR^2^(95%CI)
Q1(<23.6)	42	1(ref)	1(ref)
Q2(23.6–38.1)	92	2.49(1.68–3.68)	1.91(1.26–2.90)
Q3(38.1–61.7)	114	3.53(2.41–5.18)	2.32(1.44–3.74)
Q4(>61.7)	185	6.35(4.39–9.12)	3.31(1.76–6.25)

^1:^ crude OR by logistic regression model

^2^: logistic regression model adjusted for age, BMI, WHtR, smoking status, family history of hypertension, educational level, marital status and family income.

**Fig 1:**
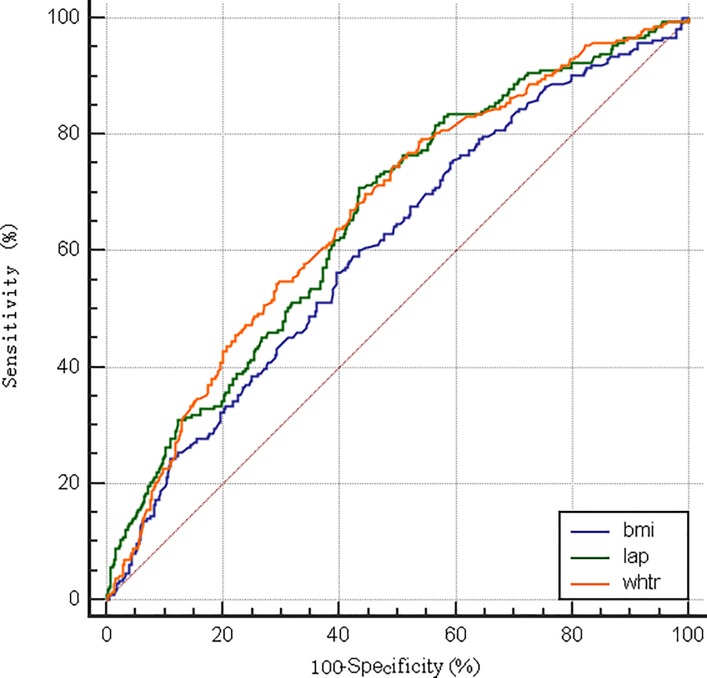
The ROC curve of different obesity indices for prediction of hypertension in males.

**Fig 2:**
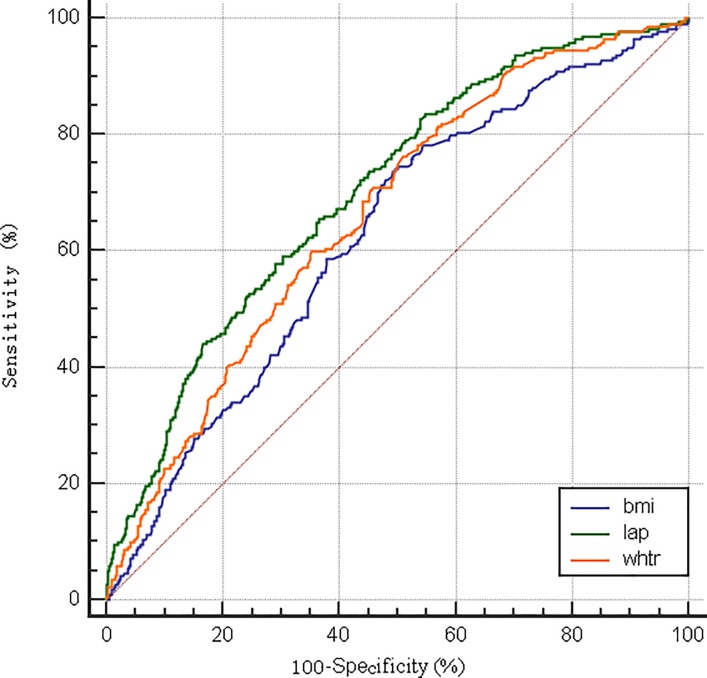
The ROC curve of different obesity indices for prediction of hypertension in females.

The AUCs and cut-off values of LAP, BMI and WHtR were presented in Table 4. The best thresholds of LAP to predict hypertension were 40.60 in male and 29.14 in female respectively. In males, the AUC (95%CI) of LAP, BMI and WHtR were 0.66 (0.62–0.69), 0.61 (0.57–0.64) and 0.67 (0.63–0.70), respectively. The AUC of LAP was significantly different with BMI (Z = 2.158, *p* = 0.0309), while not different with WHtR (Z = 0.345, *p* = 0.7305). As for females, the AUC of LAP (0.70, 95%CI: 0.67–0.73) was significantly higher than that of BMI (0.63, 95%CI: 0.60–0.66) and WHtR 0.66 (95% CI: 0.63–0.69) with *p* value of 0.0004 and 0.0303 respectively. The ROC curves were shown in Fig 1 and Fig 2.

**Table 4:** The comparisons of different obesity indices in predicting hypertension risk.

		Cut-off value	Sensitivity (%)	Specificity (%)	YI	AUC (95%CI)	*Z*	*P*^*a*^
Male	BMI	25.04	59.15	57.57	0.17	0.61(0.57–0.64)	2.158	0.0309
	WHtR	0.52	69.95	55.51	0.25	0.67(0.63–0.70)	0.345	0.7305
	LAP	40.60	70.89	56.65	0.28	0.66(0.62–0.69)	-	-
Female	BMI	24.00	73.64	50.80	0.24	0.63(0.60–0.66)	3.570	0.0004
	WHtR	0.52	76.36	49.07	0.25	0.66(0.63–0.69)	2.166	0.0303
	LAP	29.14	83.64	45.24	0.29	0.70(0.67–0.73)	-	-

^a^: AUC of BMI and WHtR, compared with that of LAP

### 3. Interactive effects analysis

Table 5 presented the results of interactive effects analysis. In males, the adjusted OR of hypertension was the highest in high-LAP and smoking subjects (3.32, 95%CI: 1.79–6.17) as compared with low-LAP and non-smoking subjects. There was a significant interaction between LAP and smoking (RERI: 1.32, 95%CI: 0.15–2.75; AP: 0.40, 95%CI: 0.14–0.73) on risk of hypertension. When analyzing the interaction between LAP and family history of history, RERI was 1.07 (95%CI: 0.09–2.05), suggesting that there would be 1.07 relative excess risk due to the interaction. AP was 0.33 (95%CI: 0.23–0.44), indicating that 33% of hypertension exposed to both risk factors was attributable to the interaction. Moreover, SI was 1.92 (95% CI: 1.53–2.41).

**Table 5:** Interactions between LAP and family history of hypertension and smoking on risk of hypertension.

Variable	Variable	Male		Female	
		OR^1^(95%CI)	Interaction effect	OR^1^(95%CI)	Interaction effect
LAP	Smoking				
-	-	1(ref)	RERI = 1.32(0.15–2.75)^2^	1(ref)	RERI = 0.04(-2.22–2.30)^3^
-	+	1.31(0.67–2.07)	AP = 0.40(0.14–0.73)^2^	1.25(0.50–3.16)	AP = 0.02(-0.89–0.92)^3^
+	-	1.69(0.89–3.21)	SI = 2.32(0.79–9.03)^3^	2.18(1.48–3.20)	SI = 1.03(0.22–4.84)^3^
+	+	3.32(1.79–6.17)		2.47(1.08–5.67)	
LAP	Family history of hypertension				
-	-	1(ref)	RERI = 1.07(0.09–2.05)^2^	1(ref)	RERI = 0.80(0.07–1.53)^2^
-	+	1.55(1.18–2.05)	AP = 0.33(0.23–0.44)^2^	1.18(0.65–2.12)	AP = 0.25(0.11–0.39)^2^
+	-	1.62(1.15–2.27)	SI = 1.92(1.53–2.41)^2^	2.17(1.45–3.24)	SI = 1.59(1.16–2.19)^2^
+	+	3.24(1.66–6.32)		3.14(1.76–5.60)	

1: adjusted for age, BMI, WHtR, smoking status, family history of hypertension, educational level, marital status and family income

2: *p*<0.05

3: *p*>0.05

In females, the adjusted OR of hypertension was also the highest in high-LAP and smoking subjects (2.47, 95%CI: 1.08–5.67) as compared with low-LAP and non-smoking subjects. However, no interactive effect between LAP and smoking was found by all three indicators. Specifically, RERI was 0.04 (95%CI:-2.22–2.30), AP was 0.02 (95%CI: -0.89–0.92) and SI was 1.03 (95%CI: 0.22–4.84). The values of RERI (0.80, 95%CI: 0.07–1.53), AP (0.25, 95%CI: 0.11–0.39) and SI (1.59, 95%CI: 1.16–2.19) indicated a significant interaction between LAP and family history of hypertension on hypertension risk.

## Discussion

In this present study, we found a significant relationship between LAP and hypertension risk in Han Chinese adults. Similar results were found in Japanese [22] and Mongolians population [23]. Furthermore, we compared the predictive value of LAP, BMI and WHtR on hypertension risk, and the results suggested that LAP was substantially better than that of BMI in both males and females, but only better than that of WHtR in females. It is probably associated with the various patterns of lipid over accumulation in both males and females with aging [35]. For women, LAP was greater at older age or remained unchanged, while for men, the annual LAP changes were reduced at older age [35]. Compared with men, hypertriglyceridemia was a stronger risk factor for cardiovascular diseases in women [36]. A cohort study suggested that LAP was a better predictor of all-cause mortality in women than men [37]. The association between LAP and diabetes risk also tended to be stronger in women than in men [38]. Therefore, LAP may be more valuable in female.

With the changes of modern lifestyle and diet patterns, the prevalence of obesity has dramatically increased in China [7]. Accumulating evidences have shown that there is a significant association of blood pressure increase with weight gain [39, 40]. The mechanisms of obesity and obesity-related hypertension are complex. Overweight and obese people can secrete more leptin, TNF-α, IL-6 and other relevant adipocytokines, which may affect endothelial cells function, renin-angiotensin system, sympathetic nervous system and inflammatory response [41, 42]. It is a remarkable fact that the function of various adipose tissues is different and complex [43, 44]. A growing number of evidences have strongly suggested that the location of fat distribution was more harmful than the total amount of fat for obese people [45, 46]. The lipolytic activity of visceral adipose tissue cells was stronger than that of subcutaneous adipose tissue cells [47]. LAP, a combination of WC and TG, was proved to be a simple and inexpensive way to assess visceral fat [13]. WC is a commonly applied obesity index to evaluate central obesity, which is proved to be associated with insulin resistance [48], all cause/cardiovascular mortality [49] and hypertension risk [50, 51]. However, WC cannot sufficiently discriminate between visceral and subcutaneous fat [52]. TG concentrations are significantly related with visceral adipose tissue. Moreover, hypertriglyceridemia was associated with an increased risk for cardiovascular diseases [53]. Rotter et al [54] studied the relationship between LAP and metabolic syndrome and its components, the results showed that LAP was significantly positively correlated with serum total cholesterol, FPG, insulin, but negatively correlated with HDL in elderly men. Therefore, the LAP that derives from WC and TG is believed to be a better predictor of hypertension risk and suitable in clinical application.

In 2000, Lemieux et al [55] introduced an index named hypertriglyceridemic waist (HTGW) that was also combined by WC and TG. The HTGW phenotype was associated with metabolic alternations and visceral fat excess [56]. 82% of individuals with HTGW phenotype had more than three cardiovascular risk factors [56]. A meta-analysis confirmed that HTGW was closely associated with increased risk of type 2 diabetes mellitus in the general population [57]. HTGW was also a better simple marker than WHtR for identifying the risk of cardiometabolic disorders [58]. A cohort study with 95015 participants in China showed that HTGW was independently associated with hypertension and cardiovascular diseases risks [59]. In comparison, HTGW is a dichotomous indicator, while LAP is developed to express a continuous risk function by gender that can better reflect the lipid accumulation and the relationship between lipid toxicity and hypertension since obesity itself is a continuous process [18]. In a cohort study, LAP, rather than HTGW, showed an association with all-cause mortality [37]. On the other hand, the cut-off values of WC and TG are controversial, and the standard of positive HTGW are not uniform. One of the advantages of LAP is that it does not arbitrarily dichotomize. According to YI index, this article showed that the cut-points of LAP were higher in men than those in women, which was coherent with previous study [37].

According to our results, family history of hypertension was significantly interacted with LAP on hypertension risk both in males and females. As hypertension is a multifactorial disease that associated with genetic and environmental factors, the interaction between genes and the environment may aggravate the risk of hypertension. A cross-sectional survey showed that the prevalence of hypertension in adults with family history of hypertension was 29.3% and 24.4% in adults without family history of hypertension [60]. Also, several studies have shown that family history of hypertension was positively associated with the risk of overweight either in children or adults [61, 62].

Our results showed that smoking was interacted with LAP on hypertension risk in males while not in females. It is the fact that the smoking rate is very low in women but very high in men especially in middle-aged and elderly adults in China [63]. In this study, the smoking rate was 61.2% in males and 6.61% in females, respectively. A cohort study reported a significant interaction between smoking with abdominal obesity on diabetes risk in Chinese adults, but not with overall obesity [64]. Similarly, Cullen et al [65] reported a non-statistically significant interaction of smoking and BMI on diabetes risk in elderly women. Previous researches have indicated that smoking represented a major health hazard, which was an important risk factor for cardiovascular diseases. The mechanisms of interactive effect between visceral obesity and smoking on hypertension risk may be explained by the elevation of blood pressure levels via inhibiting vascular reflex vasodilation and damaging vascular endothelial function [66]. It should be noted that numerous Chinese non-smokers are exposed to second environmental tobacco smoke. A published meta-analysis has concluded that the pooled prevalence of passive smoking in the community population aged 15 years and older female in China were 47.8% [67]. Moreover, exposure to secondhand smoke frequently was significantly related with hypertension among nonsmoking female in China [68, 69]. A study included 5027731 females along with their husbands in 31 provinces in China confirmed that cumulative exposure of husband smoking was significantly associated with the risk of hypertension for females [70]. Unfortunately, the information about passive smoking of female was not investigated in this survey. Future research should be carried out to detect interactions between passive smoke of females and obesity, which may further light on the etiology of hypertension.

There are several published studies exploring the application value of LAP in diseases prediction. Dai et al [11] compared the ability of different obesity indices in predicting chronic kidney disease among the rural population in Northeast China, and the results showed that LAP performed better than that of BMI, WC and WHtR. Compared with BMI, LAP was proved to be a better predictor in the incidence of cardiovascular diseases [18]. LAP levels were independently associated with all-cause, cardiovascular and congestive heart failure mortality in normal weight postmenopausal women, whereas no significant associations were found in men [71]. In polycystic ovary syndrome women, the AUC of LAP was significantly higher than BMI and WC when compared the ability in predicting impaired glucose tolerance [20]. Meanwhile, LAP had a greater impact on the homeostasis model assessment of insulin resistance (HOMA-IR) than BMI and WC by multivariate analysis [72]. Chiang et al [73] tested the accuracy of LAP in predicting metabolic syndrome in middle-aged and elderly Taiwanese adults in China, and LAP was proved to be a simple index with significantly higher predictability. The similar results were found in Iran population [74]. A population based cohort study among Iran adults suggested that LAP was an independent predictor of cardiovascular events in normal BMI subjects [12].

Several limitations in this study needed to be pointed out. Firstly, it was a cross- sectional study, which cannot infer causality of our results. Secondly, the lack of information on the use of lipid-lowering drugs may influence the results. Thirdly, the participants in this study were all middle-aged and elderly. A study in Japan found that the association between LAP and diabetes risk were influenced by age [38]. The association between LAP and hypertension risk in younger groups may need to be further explored.

## Conclusion

In conclusion, it is crucial to assess visceral fat accumulation in a convenient and cheap way for the prevention of cardiovascular diseases. Our study suggests that LAP is significantly associated with hypertension risk and performed better than that of other obesity indices. As traditional assessment methods of visceral fat evaluation are not available in daily clinical application, LAP can be extensively used in epidemiological studies and some large-scale clinical trials. Moreover, this is the first study that further demonstrates interactive effects of LAP and smoking, family history of hypertension on hypertension risk.

## Supporting information

S1 File Survey questionnaire in Chinese. (DOCX)

S2 File Survey questionnaire in English. (DOCX)

## Abbreviations

BMI: body mass index
WHtR: waist-to-height ratio
WC: Waist circumference
FPG: fasting plasma glucose
TG: triglycerides
LAP: lipid accumulation product

## References

[1] Campbell NR, Lackland DT, Niebylski ML, World Hypertension League Committee, International Society of Hypertension Executive Committee. High blood pressure: why prevention and control are urgent and important: a 2014 fact sheet from the World Hypertension League and the International Society of Hypertension [J]. J Clin Hypertens (Greenwich).2014, 16(8):551–3. 25040331 10.1111/jch.12372 PMC8032157

[2] Li Y, Yang L, Wang L, Zhang M, Huang Z, Deng Q. Burden of hypertension in China: A nationally representative survey of 174,621 adults [J]. Int J Cardiol. 2016, 227:516–23. doi: 10.1016/j.ijcard.2016.10.110 27856040 10.1016/j.ijcard.2016.10.110

[3] Bundy JD, He J. Hypertension and Related Cardiovascular Disease Burden in China [J]. Ann Glob Health. 2016, 82(2):227–33. doi: 10.1016/j.aogh.2016.02.002 27372527 10.1016/j.aogh.2016.02.002

[4] Lewington S, Lacey B, Clarke R, Guo Y, Kong XL, Yang L, et al The Burden of Hypertension and Associated Risk for Cardiovascular Mortality in China [J]. JAMA Intern Med. 2016,176 (4): 524–32. doi: 10.1001/jamainternmed.2016.0190 26975032 10.1001/jamainternmed.2016.0190

[5] Yatsuya H, Li Y, Hilawe EH, Ota A, Wang C, Chiang C, et al Global trend in overweight and obesity and its association with cardiovascular disease incidence [J]. Circ J. 2014, 78(12):2807–18. 25391910 10.1253/circj.cj-14-0850

[6] Seravalle G, Grassi G. Obesity and hypertension [J]. Pharmacol Res. 2017, 122: 1–7. doi: 10.1016/j.phrs.2017.05.013 28532816 10.1016/j.phrs.2017.05.013

[7] Wu J, Xu H, He X, Yuan Y, Wang C, Sun J, et al Six-year changes in the prevalence of obesity and obesity- related diseases in Northeastern China from 2007 to 2013 [J]. Sci Rep.2017, 7:41518. 10.1038/srep41518 PMC5269745 28128316

[8] Carmienke S, Freitag MH, Pischon T, Schlattmann P, Fankhaenel T, Goebel H, et al General and abdominal obesity parameters and their combination in relation to mortality: a systematic review and meta-regression analysis [J]. Eur J Clin Nutr. 2013, 67(6):573–85. doi: 10.1038/ejcn.2013.61 23511854 10.1038/ejcn.2013.61

[9] Mi SQ, Yin P, Hu N, Li JH, Chen XR, Chen B, et al BMI, WC, WHtR, VFI and BFI: which indictor is the most efficient screening index on type 2 diabetes in Chinese community population [J]. Biomed Environ Sci. 2013, 26(6):485–91. doi: 10.3967/0895-3988.2013.06.009 23816582 10.3967/0895-3988.2013.06.009

[10] Caminha TC, Ferreira HS, Costa NS, Nakano RP, Carvalho RE, Xavier AF Jr, et al Waist-to-height ratio is the best anthropometric predictor of hypertension: A population-based study with women from a state of northeast of Brazil [J]. Medicine (Baltimore). 2017, 96(2):e5874. 28079826 10.1097/MD.0000000000005874 PMC5266188

[11] Dai D, Chang Y, Chen Y, Chen S, Yu S, Guo X et al Visceral Adiposity Index and Lipid Accumulation Product Index: Two Alternate Body Indices to Identify Chronic Kidney Disease among the Rural Population in Northeast China [J]. Int J Environ Res Public Health. 2016, 13(12). pii:E1231. 10.3390/ijerph13121231 PMC5201372 27983609

[12] Hosseinpanah F, Barzin M, Mirbolouk M, Abtahi H, Cheraghi L, Azizi F. Lipid accumulation product and incident cardiovascular events in a normal weight population: Tehran Lipid and Study [J]. Eur J Prev Cardiol. 2016 23 (2):187–93. doi: 10.1177/2047487314558771 25381336 10.1177/2047487314558771

[13] Sandeep S, Gokulakrishnan K, Velmurugan K, Deepa M, Mohan V. Visceral & subcutaneous abdominal fat in relation to insulin resistance & metabolicsyndrome in non-diabetic south Indians [J]. Indian J Med Res. 2010, 131:629–35. 20516533

[14] Schlecht I, Gronwald W, Behrens G, Baumeister SE, Hertel J, Hochrein J, et al Visceral adipose tissue but not subcutaneous adipose tissue is associated with urine and serum metabolites [J]. PLoS One. 2017, 12(4):e0175133 doi: 10.1371/journal.pone.0175133 28403191 10.1371/journal.pone.0175133 PMC5389790

[15] Giacchetti G, Faloia E, Mariniello B, Sardu C, Gatti C, Camilloni MA, et al Overexpression of the renin- angiotensin system in human visceral adipose tissue in normal and overweight subjects [J]. Am J Hypertens. 2002, 15(5):381–8. 12022238 10.1016/s0895-7061(02)02257-4

[16] Atzmon G, Yang XM, Muzumdar R, Ma XH, Gabriely I, Barzilai N. Differential gene expression between visceral and subcutaneous fat depots [J]. Horm Metab Res. 2002, 34:622–8. doi: 10.1055/s-2002-38250 12660871 10.1055/s-2002-38250

[17] Alvarez GE, Beske SD, Ballard TP, Davy KP. Sympathetic neural activation in visceral obesity [J]. Circulation. 2002, 106:2533–6. 12427647 10.1161/01.cir.0000041244.79165.25

[18] Kahn HS. The "lipid accumulation product" performs better than the body mass index for recognizing cardiovascular risk: a population-based comparison [J]. BMC Cardiovasc Disord. 2005, 5:26 doi: 10.1186/1471-2261-5-26 16150143 10.1186/1471-2261-5-26 PMC1236917

[19] Xia C, Li R, Zhang S, Gong L, Ren W, Wang Z, et al Lipid accumulation product is a powerful index for recognizing insulin resistance in non-diabetic individuals [J]. Eur J Clin Nutr.2012 66(9):1035–8. doi: 10.1038/ejcn.2012.83 22781025 10.1038/ejcn.2012.83

[20] Wehr E, Gruber HJ, Giuliani A, Moller R, Pieber TR, Obermayer-Pietsch B. The lipid accumulation product is associated with impaired glucose tolerance in PCOS women [J]. J Clin Endocrinol Metab. 2011 96(6):E986–90. doi: 10.1210/jc.2011-0031 21470992 10.1210/jc.2011-0031

[21] Kahn HS. The lipid accumulation product is better than BMI for identifying diabetes: a population-based comparison [J].Diabetes Care.2006, 29(1):151–3 16373916 10.2337/diacare.29.1.151

[22] Wakabayashi I. Associations of blood lipid-related indices with blood pressure and pulse pressure in middle-aged men [J]. Metab Syndr Relat Disord. 2015, 13 (1): 22–8. doi: 10.1089/met.2014.0093 25321638 10.1089/met.2014.0093

[23] Gao X, Wang G, Wang A, Xu T, Tong W, Zhang Y. Comparison of lipid accumulation product with body mass index as an indicator of hypertension risk among Mongolians in China [J]. Obes Res Clin Pract. 2013, 7(4):e308–14 doi: 10.1016/j.orcp.2012.02.002 24306160 10.1016/j.orcp.2012.02.002

[24] Nazare JA, Smith JD, Borel AL, Haffner SM, Balkau B, Ross R, et al Ethnic influences on the relations between abdominal subcutaneous and visceral adiposity, liver fat, and cardiometabolic risk profile: the International Study of Prediction of IntraAbdominal Adiposity and Its Relationship with Cardiometabolic Risk/ IntraAbdominal Adiposity [J]. Am J Clin Nutr. 2012, 96:714–26. doi: 10.3945/ajcn.112.035758 22932278 10.3945/ajcn.112.035758

[25] Virdis A, Giannarelli C, Neves MF, Taddei S, Ghiadoni L. Cigarette smoking and hypertension [J]. Curr Pharm Des. 2010, 16(23):2518–25. 20550499 10.2174/138161210792062920

[26] Igarashi R, Fujihara K, Heianza Y, Ishizawa M, Kodama S, Saito K, et al Impact of individual components and their combinations within a family history of hypertension on the incidence of hypertension: Toranomon hospital health management center study 22 [J]. Medicine (Baltimore). 2016, 95(38):e4564. 27661014 10.1097/MD.0000000000004564 PMC5044884

[27] Chobanian AV, Bakris GL, Black HR, Cushman WC, Green LA, Izzo JL Jr, et al The seventh report of the joint national committee on prevention, detection, evaluation, and treatment of high blood pressure: the JNC 7 report [J]. JAMA.2003, 289: 2560–72. doi: 10.1001/jama.289.19.2560 12748199 10.1001/jama.289.19.2560

[28] Alberti KG, Zimmet PZ. Definition, diagnosis and classification of diabetes mellitus and its complications. Part 1: diagnosis and classification of diabetes mellitus provisional report of a WHO consultation [J]. Diabet Med. 1998, 15:539–53. doi: 10.1002/(SICI)1096-9136(199807)15:7<539::AID-DIA668>3.0.CO;2-S 9686693 10.1002/(SICI)1096-9136(199807)15:7<539::AID-DIA668>3.0.CO;2-S

[29] Joint Committee for Developing Chinese guidelines on Prevention and Treatment of Dyslipidemia in Adults. Chinese guidelines on prevention and treatment of dyslipidemia in adults [J]. Zhonghua Xin Xue Guan Bing Za Zhi. 2007, 35(5):390–419. (In Chinese) 17711682

[30] Writing Group of 2010 Chinese Guidelines for the Management of Hypertension. 2010 Chinese guidelines for the management of hypertension [J]. Chine J Cardiology 2011, 39:579–616. (In Chinese) 22088239

[31] Chen C, Lu FC, Department of Disease Control Ministry of Health, PR China. The guidelines for prevention and control of overweight and obesity in Chinese adults [J]. Biomed EnvironSci. 2004, 17 Suppl:1–36. 15807475

[32] Browning LM, Hsieh SD, Ashwell M. A systematic review of waist to-height ratio as a screening tool for the prediction of cardiovascular disease and diabetes: 0.5 could be a suitable global boundary value [J]. Nutr Res Rev.2010,23(2):247–69. doi: 10.1017/S0954422410000144 20819243 10.1017/S0954422410000144

[33] Andersson T, Alfredsson L, Kallberg H, Zdravkovic S, Ahlbom A. Calculating measures of biological interaction [J]. Eur J Epidemiol. 2005, 20:575–9. 16119429 10.1007/s10654-005-7835-x

[34] Knol MJ, VanderWeele TJ, Groenwold RH, Klungel OH, Rovers MM, Grobbee DE. Estimating measures of interaction on an additive scale for preventive exposures [J]. Eur J Epidemiol. 2011, 26 (6): 433–38. doi: 10.1007/s10654-011-9554-9 21344323 10.1007/s10654-011-9554-9 PMC3115067

[35] Kahn HS, Cheng YJ. Longitudinal changes in BMI and in an index estimating excess lipids among white and black adults in the United States [J]. Int J Obes (Lond) 2008, 32:136–43. 17684512 10.1038/sj.ijo.0803697

[36] Hokanson JE, Austin MA. Plasma triglyceride level is a risk factor for cardiovascular disease independent of high-density lipoprotein cholesterol level: a meta-analysis of population-based prospective studies [J]. J Cardiovasc Risk. 1996 3: 213–19. 8836866

[37] Ioachimescu AG, Brennan DM, Hoar BM, Hoogwerf BJ. The Lipid Accumulation Product and All-cause Mortality in Patients at High Cardiovascular Risk: A PreCIS Database Study [J]. Obesity (Silver Spring). 2010, 18(9):1836–44. 20035284 10.1038/oby.2009.453

[38] Wakabayashi I. Influence of age and gender on lipid accumulation product and its relation to diabetes mellitus in Japanese [J]. Clin Chim Acta. 2014, 431:221–6. doi: 10.1016/j.cca.2014.02.002 24530297 10.1016/j.cca.2014.02.002

[39] Zhou L, Li Y, Guo M, Wu Y, Zhao L. Relations of body weight status in early adulthood and weight changes until middle age with hypertension in the Chinese population [J]. Hypertens Res. 2016, 39(12):913–8. doi: 10.1038/hr.2016.80 27357058 10.1038/hr.2016.80

[40] Ren Q, Su C, Wang H, Wang Z, Du W, Zhang B. Change in Body Mass Index and Its Impact on Incidence of Hypertension in 18-65-Year-Old Chinese Adults[J]. Int J Environ Res Public Health.2016, 13(3). pii: E257. 10.3390/ijerph13030257 PMC4808920 26927144

[41] Seravalle G, Grassi G. Obesity and hypertension [J]. Pharmacol Res. 2017, 122:1–7. doi: 10.1016/j.phrs.2017.05.013 28532816 10.1016/j.phrs.2017.05.013

[42] Seravalle G, Grassi G. Sympathetic Nervous System, Hypertension, Obesity and Metabolic Syndrome [J]. High Blood Press Cardiovasc Prev. 2016, 23(3):175–9. doi: 10.1007/s40292-016-0137-4 26942609 10.1007/s40292-016-0137-4

[43] Frayn KN, Karpo F, Fielding BA, Macdonald IA, Coppack SW. Integrative physiology of human adipose tissue [J]. Int J Obes Relat Metab Disord. 2003, 27:875–88. doi: 10.1038/sj.ijo.0802326 12861227 10.1038/sj.ijo.0802326

[44] Karelis AD, St Pierre DH, Conus F, Rabasa-Lhoret R, Poehlman ET. Metabolic and body composition factors in subgroups of obesity: what do we know? [J]. J Clin Endoerinol Metab.2004, 89: 2569–75. 10.1210/jc.2004-0165 15181025

[45] Li Y, Bujo H, Takahashi K, Shibasaki M, Zhu Y, Yoshida Y, et al Visceral fat: higher responsiveness of fat mass and gene expression to calorie restriction thansubcutaneous fat[J]. Exp Biol Med (Maywood).2003, 228(10):1118–23. 14610249 10.1177/153537020322801004

[46] Hoffstedt J, Arner E, Wahrenberg H, Andersson DP, Qvisth V, Lofgren P, et al Regional impact of adipose tissue morphology on the metabolic profile in morbid obesity[J]. Diabetologia.2010, 53 (12): 2496–2503. doi: 10.1007/s00125-010-1889-3 20830466 10.1007/s00125-010-1889-3

[47] Lima-Martinez MM, Blandenier C, Iacobellis G. Epicardial adipose tissue: more than a simple fat deposit?[J]. Endocrinol Nutr. 2013, 60(6):320–28. doi: 10.1016/j.endonu.2012.08.001 23117053 10.1016/j.endonu.2012.08.001

[48] Wolfgram PM, Connor EL, Rehm JL, Eickhoff JC, Zha W, Reeder SB, et al In Nonobese Girls, Waist Circumference as a Predictor of Insulin Resistance Is Comparable to MRI Fat Measures and Superior to BMI [J]. Horm Res Paediatr. 2015, 84(4):258–65. doi: 10.1159/000439130 26352642 10.1159/000439130 PMC4644098

[49] David CN, Mello RB, Bruscato NM, Moriguchi EH. Overweight and Abdominal Obesity Association with All-Cause and Cardiovascular Mortality in the Elderly Aged 80 and Over: A Cohort Study [J]. J Nutr Health Aging. 2017 21(5):597–603. doi: 10.1007/s12603-016-0812-0 28448093 10.1007/s12603-016-0812-0 PMC12878212

[50] Nurdiantami Y, Watanabe K, Tanaka E, Pradono J, Anme T, et al Association of general and central obesity with hypertension [J]. Clin Nutr. 2017 pii:S0261- 5614(17):30173–5. 10.1016/j.clnu.2017.05.012 28583324

[51] Sun H, Zheng M, Wu S, Chen M, Cai J, Yang X. Waist circumference and incidence of hypertension in Chinese adults: Observations from the Kailuan Study[J]. Herz. 2016. 10.1007/s00059-016-4501-x 27928596

[52] Du T, Sun X, Huo R, Yu X. Visceral adiposity index, hypertriglyceridemic waist and risk of diabetes: the China Health and Nutrition Survey 2009 [J]. Int J Obes (Lond). 2014 38(6):840–7. 24048141 10.1038/ijo.2013.181

[53] Kim EH, Lee JB, Kim SH, Jo MW, Hwang JY, Bae SJ, et al Serum Triglyceride Levels and Cardiovascular Disease Events in Koreans [J]. Cardiology. 2015, 131 (4): 228–35. doi: 10.1159/000380941 25968991 10.1159/000380941

[54] Rotter I, Ryl A, Szylinska A, Pawlukowska W 1, Lubkowska A, Laszczynska M. Lipid Accumulation Product (LAP) as an Index of Metabolic and Hormonal Disorders in Aging Men[J].Exp Clin Endocrinol Diabetes. 2017 125(3):176–82. doi: 10.1055/s-0042-116071 27898988 10.1055/s-0042-116071

[55] Lemieux I, Pascot A, Couillard C, Lamarche B, Tchernof A, Almeras N, et al Hypertriglyceridemic waist: A marker of the atherogenic metabolic triad (hyperinsulinemia; hyperapolipoprotein B; small, dense LDL) in men? [J]. Circulation. 2000, 102(2):179–84. 10889128 10.1161/01.cir.102.2.179

[56] Cunha de Oliveira C, Carneiro Roriz AK, Eickemberg M, Barreto Medeiros JM, Barbosa Ramos L. Hypertriglyceridemic waist phenotype: association with metabolic disorders and visceral fat in adults [J]. Nutr Hosp. 2014, 30(1):25–31. doi: 10.3305/nh.2014.30.1.7411 25137258 10.3305/nh.2014.30.1.7411

[57] Ren Y, Luo X, Wang C, Yin L, Pang C, Feng T, et al Prevalence of hypertriglyceridemic waist and association with risk of type 2 diabetes mellitus: a meta-analysis [J]. Diabetes Metab Res Rev. 2016, 32(4):405–12. doi: 10.1002/dmrr.2725 26417844 10.1002/dmrr.2725

[58] Bailey DP, Savory LA, Denton SJ, Davies BR, Kerr CJ. The hypertriglyceridemic waist, waist-to-height ratio, and cardiometabolic risk [J]. J Pediatr.2013, 162 (4):746–52 doi: 10.1016/j.jpeds.2012.09.051 23140880 10.1016/j.jpeds.2012.09.051

[59] Wang A, Li Z, Zhou Y, Wang C, Luo Y, Liu X, et al Hypertriglyceridemic waist phenotype and risk of cardiovascular diseases in China: results from the Kailuan Study [J]. Int J Cardiol. 2014, 174(1):106–9 doi: 10.1016/j.ijcard.2014.03.177 24745860 10.1016/j.ijcard.2014.03.177

[60] Ranasinghe P, Cooray DN, Jayawardena R, Katulanda P. The influence of family history of Hypertension on disease prevalence and associated metabolic risk factors among Sri Lankan adults [J]. BMC Public Health. 2015 15:576 doi: 10.1186/s12889-015-1927-7 26092387 10.1186/s12889-015-1927-7 PMC4475303

[61] Liu J, Sekine M, Tatsuse T, Hamanishi S, Fujimura Y, Zheng X. Family history of hypertension and the risk of overweight in Japanese children: results from the Toyama Birth Cohort Study [J]. J Epidemiol. 2014, 24(4):304–11. doi: 10.2188/jea.JE20130149 24857956 10.2188/jea.JE20130149 PMC4074635

[62] Ranasinghe P, Cooray DN, Jayawardena R, Katulanda P. The influence of family history of hypertension on disease prevalence and associated metabolic risk factors among Sri Lankan adults [J]. BMC Public Health. 2015,15:576 doi: 10.1186/s12889-015-1927-7 26092387 10.1186/s12889-015-1927-7 PMC4475303

[63] Yang GH, Li Q, Wang CX, Hsia J, Yang Y, Xiao L, et al Findings from 2010 Global Adult Tobacco Survey: implementation of MPOWER policy in China [J]. Biomed Environ Sci. 2010, 23:422–9. 10.1016/S0895-3988(11)60002-0 21315239

[64] Luo W, Guo Z, Wu M, Hao C, Zhou Z, Yao X, et al Interaction of smoking and obesity on type 2 diabetes risk in a Chinese cohort [J]. Physiol Behav. 2015, 139: 240–3. doi: 10.1016/j.physbeh.2014.11.038 25449404 10.1016/j.physbeh.2014.11.038

[65] Cullen MW, Ebbert JO, Vierkant RA, Wang AH, Cerhan JR. No interaction of body mass index and smoking on diabetes mellitus risk in elderly women [J]. Prev Med. 2009,48(1):74–8. doi: 10.1016/j.ypmed.2008.10.008 19000710 10.1016/j.ypmed.2008.10.008 PMC2664829

[66] Talukder MAH, Johnson WM, Varadharaj S, Lian J, Kearns PN, El-Mahdy MA, et al Chronic cigarette smoking causes hypertension, increased oxidative stress, impaired NO bioavailability, endothelial dysfunction, and cardiac remodeling in mice [J]. Am J Physiol Heart Circ Physiol. 2011, 300(1):388–96 10.1152/ajpheart.00868.2010 PMC3023256 21057039

[67] Zeng J, Yang S, Wu L, Wang J, Wang Y, Liu M, et al Prevalence of passive smoking in the community population aged 15 years and older in China: a systematic review and meta-analysis [J]. BMJ Open. 2016,6:e009847 doi: 10.1136/bmjopen-2015-009847 27059465 10.1136/bmjopen-2015-009847 PMC4838695

[68] Li N, Li Z, Chen S, Yang N, Ren A, Ye R. Effects of passive smoking on hypertension in rural Chinese nonsmoking women[J]. J Hypertens. 2015,33(11): 2210–4. doi: 10.1097/HJH.0000000000000694 26259123 10.1097/HJH.0000000000000694

[69] Wu L, Yang S, He Y, Liu M, Wang Y, Wang J, et al Association between passive smoking and hypertension in Chinese non-smoking elderly women[J]. Hypertens Res. 2017,40(4):399–404. doi: 10.1038/hr.2016.162 27928149 10.1038/hr.2016.162

[70] Yang Y, Liu F, Wang L, Li Q, Wang X, Chen JC, et al Association of Husband Smoking With Wife’s Hypertension Status in Over 5 Million Chinese Females Aged 20 to 49 Years [J].J Am Heart Assoc. 2017,6(3). pii: e004924. 10.1161/JAHA.116.004924 PMC5524022 28320748

[71] Wehr E, Pilz S, Boehm BO, Marz W, Obermayer-Pietsch B. The Lipid Accumulation Product Is Associated With Increased Mortality in Normal Weight Postmenopausal Women [J]. Obesity (Silver Spring). 2011, 19(9):1873–80. 21394091 10.1038/oby.2011.42

[72] Xia C, Li R, Zhang S, Gong L, Ren W, Wang Z, et al Lipid accumulation product is a powerful index for recognizing insulin resistance in non-diabetic individuals [J]. Eur J Clin Nutr. 2012 66(9):1035–8. doi: 10.1038/ejcn.2012.83 22781025 10.1038/ejcn.2012.83

[73] Chiang JK, Koo M. Lipid accumulation product: a simple and accurate index for predicting metabolic syndrome in Taiwanese people aged 50 and over [J].BMC Cardiovasc Disord. 2012, 24(12):78. 10.1186/1471-2261-12-78 PMC3506496 23006530

[74] Motamed N, Razmjou S, Hemmasi G, Maadi, Zamani F. Lipid accumulation product and metabolic syndrome: a population-based study in northern Iran, Amol [J]. J Endocrinol Invest. 2016, 39(4):375–82. doi: 10.1007/s40618-015-0369-5 26319991 10.1007/s40618-015-0369-5

