# Supplementary material for: The effect of lipid accumulation product and its interaction with other factors on hypertension risk in Chinese Han population: A cross-sectional study
Source: PLoS One. 2018 Jun 6;13(6):e0198105. doi: 10.1371/journal.pone.0198105 (PMC5991403; doi:10.1371/journal.pone.0198105)
Supplement: S1 File — (DOCX) [file pone.0198105.s001.docx]

调查问卷

A1. 住户成员姓名：

家庭住址：

A2. 您的年龄： 周岁（出生日期 年 月，以身份证上信息为准）

A3. 性别： 1.男 2.女

A4. 您的文化程度？

1.小学学历及以下 2.初中学历 3.高中学历及以上(包括中专/技校)

A5. 您目前的婚姻状况？

1.目前未婚(包括离婚，丧偶) 2.目前已婚

A6.您的家庭月收入？

1.0-2000 2.2000-4000 3. 4000-

A7. 您是否有高血压家族病史？（可多选）

1.是 2.否

A8.您现在吸烟吗？________

1.吸 2.以前吸，但现在不吸 3.从不吸烟
