# Supplementary material for: The effect of lipid accumulation product and its interaction with other factors on hypertension risk in Chinese Han population: A cross-sectional study
Source: PLoS One. 2018 Jun 6;13(6):e0198105. doi: 10.1371/journal.pone.0198105 (PMC5991403; doi:10.1371/journal.pone.0198105)
Supplement: S2 File — (DOCX) [file pone.0198105.s002.docx]

**Questionnaire**

**A1.** Name：

Home address：

**A2.** Your age： years old（birth data： year month, based on the information on the ID card）

**A3.** Gender： 1. Male 2.Female

**A4.** Your educational level?

1. Elementary school or lower 2. Middle school graduate 3. High school graduate or higher (including special secondary school and technical school)

**A5.** Your current marital status?

1. Currently not married (including divorce and widowhood)

2. Currently not married

**A6.** Your family income monthly？

1. 0-2000 2. 2000-4000 3. 4000-

**A7.** Do you have a family history of hypertension？

1. Yes 2. No

**A8.** Do you smoke？________

1. Current-smoking 2. Pre-smoking 3. Never-smoking
